# Optimizing pain management and pupil dilation in cataract surgery: a systematic review and meta-analysis of phenylephrine/ketorolac (OMIDRIA®)

**DOI:** 10.1007/s00417-025-06811-y

**Published:** 2025-03-29

**Authors:** Mohamed Abo Zeid, Amr Elrosasy, Kareem Khalefa, Mohamed Elhadary, Shrouk F. Mohamed, Amr Elkelany, Hashem Abu Serhan

**Affiliations:** 1 Faculty of Medicine, Tanta University, Tanta, Egypt; 2 Faculty of Medicine, Cairo University, Cairo, Egypt; 3 College of Medicine, QU Health, Qatar University, 2713 Doha, Qatar; 4 Faculty of Medicine, Alexandria University, Alexandria, Egypt; 5 Medical Research Group of Egypt (MRGE), Negida Academy, Arlington, MA USA; 6 Department of Ophthalmology, Hamad Medical Corporation, Doha, Qatar

**Keywords:** Cataract surgery, Ketorolac, Phenylephrine, Postoperative pain, Pupil dilation

## Abstract

**Purpose:**

This systematic review and meta-analysis aims to evaluate the efficacy and safety of the combination of phenylephrine 1% and ketorolac 0.3% (OMIDRIA®) for optimizing pain management and maintaining pupil dilation during cataract surgery. Comparisons were made against placebo/vehicle, phenylephrine alone, and epinephrine.

**Methods:**

A comprehensive search of PubMed, Cochrane CENTRAL, Embase, Scopus, and Web of Science was conducted. Eligible studies were randomized clinical trials and observational studies assessing intracameral phenylephrine/ketorolac against control groups. Key outcomes included pain management, pupil diameter, and adverse events. Data were synthesized using meta-analysis with fixed and random-effects models, and heterogeneity was assessed using the I^2^ statistic.

**Results:**

Ten studies, including 220,061 patients, were analyzed. The combination of phenylephrine/ketorolac significantly reduced postoperative pain (RR = 0.72, 95% CI: 0.60–0.86) and opioid use (RR = 0.45, 95% CI: 0.23–0.89) compared to vehicle and epinephrine. PE/K also maintained a larger pupil diameter (MD = 0.54 mm, 95% CI: 0.32–0.75) with minimal heterogeneity (I^2^ = 0%) and reduced the incidence of severe pain (RR = 0.41, 95% CI: 0.27–0.63). No significant differences in adverse events such as elevated intraocular pressure, inflammation, or headaches were observed.

**Conclusion:**

Phenylephrine/ketorolac (OMIDRIA®) demonstrates superior efficacy in maintaining intraoperative mydriasis, reducing postoperative pain, and minimizing opioid use without increasing adverse events. This combination offers a preferable alternative to traditional agents, potentially setting a new standard for pain management and pupil dilation in cataract surgery.

**Supplementary Information:**

The online version contains supplementary material available at 10.1007/s00417-025-06811-y.

## Key messages

*****What is known***:**

- Phenylephrine and ketorolac effectively maintain pupil size and reduce postoperative pain during cataract surgery, as demonstrated in previous controlled clinical trials.

*****What is new***:**

- The combination of phenylephrine/ketorolac significantly reduces the need for postoperative pain medications, including opioids, compared to placebo/vehicle and epinephrine.
- Phenylephrine/ketorolac maintains superior intraoperative mydriasis (pupil dilation) compared to other agents, reducing the risk of complications during cataract surgery.
- Despite its effectiveness in maintaining mydriasis and reducing pain, phenylephrine/ketorolac does not increase the risk of adverse events such as elevated intraocular pressure, ocular inflammation, or headaches compared to alternative treatments.

**Supplementary Information:**

The online version contains supplementary material available at 10.1007/s00417-025-06811-y.

## Introduction

Even though cataracts are virtually treatable, they remain one of the most prevalent causes of vision impairment globally lowering a patient’s quality of life. Accounting for 12 million of 36 million blind people worldwide [1], the National Eye Institute predicts that about at by 2050, this number will double. Over 50% of Americans over 75 will have been diagnosed with cataracts [2].

Most cataracts are age-related, while some may be congenital, trauma-related, or drug-induced. Cataract surgery is the therapy of choice, regardless of the etiology [3]. It developed from couching and extracapsular cataract extraction (ECCE) to phacoemulsification and intraocular lenses which use ultrasound to emulsify the lenses followed by suctioning out [4]. But the probability of improper instrument handling intraoperative and post operative pain may present which may lead to sudden eye movements and squeezing that increase the risk of intraoperative complications, postoperative pain during the initial hours following surgery was common and declined following hospital release [5, 6].

Anesthesiologists frequently use opioids like fentanyl to monitor patients during cataract surgery. Opioids provide greater pain relief and increase patient satisfaction by increasing patient comfort [5, 7]. Omidria® (Omeros Corporation, Seattle, WA, USA) is a combination medication that is continuously administered intracamerally during cataract surgery. It contains a mydriatic agent (1% phenylephrine) and an NSAID (0.3% ketorolac). Controlled clinical trials have demonstrated its effectiveness in preventing intraoperative miosis and minimizing postoperative eye discomfort [5, 8]. Mydriatics like phenylephrine help with miosis which is another aspect of interest in cataract surgery with the potential to increase the risk of complications and surgery failure, while ketorolac reduces prostaglandin synthesis thus improving pain [2, 9].

Currently, several intracameral medications are utilized during cataract surgery; however, the U.S. Food and Drug Administration (FDA) has only approved one combination agent, OMIDRIA (phenylephrine 1%/ketorolac 0.3%), to be used during cataract surgery to minimize postoperative ocular pain and maintain pupil size by preventing intraoperative miosis [10, 11].

This systematic review and meta-analysis was powered to assess the overall safety and efficacy of OMIDRIA on intra and post-operative pain management associated with miosis in cataract surgeries compared to either drug alone (like phenylephrine or epinephrine) or vehicle in adults and children undergoing phacoemulsification.

## Methods

This study was carried out according to the Preferred Reporting Items for Systematic Reviews and Meta-Analyses (PRISMA) [12] and was prospectively registered on Open Science Framework (OSF) (registration 10.17605/OSF.IO/WMX82).

### Search strategy

Databases such as PubMed, Cochrane CENTRAL, Embase, Scopus, and Web of Science (WOS) were searched for relevant studies using the following search strategy: ((Phenylephrine OR "Neo Synephrine" OR Neosynephrine OR Metaoxedrin OR Metasympatol OR Mezaton OR epinephrine OR Adrenaline OR Epifrin OR Epitrate) AND (placebo OR vehicle OR saline OR Ketorolac OR "Ketorolac tromethamine" OR Toradol) AND (Cataract OR Cataracts OR "Lens Opacities" OR "Lens Opacity" OR Pseudoaphakia OR "Cataract Extraction" OR Phakectomy OR phacoemulsification)).

### Eligibility criteria

We formulated the search query according to the PICO framework:

- Language: Studies published in English.
- Population: Patients who had cataract surgery.
- Intervention: Intracameral phenylephrine plus ketorolac (PE/K).
- Comparator: Placebo or vehicle or phenylephrine or epinephrine.
- Outcome measures: pain management, pupil diameter, ocular pain, intra-ocular pressure, best-corrected visual acuity, photophobia, and adverse events.
- Study design: All randomized clinical trials, and observational studies either case–control or cohort studies.

We excluded reviews, case reports, editorial letters, conference abstracts and study protocols, animal studies, and studies with insufficient data.

### Study selection and data extraction

After the process of screening the titles and abstracts, the full-text articles were evaluated. This selection was carried out independently by two investigators according to the criteria of inclusions and exclusions using Rayyan website [13]. Two reviewers also performed data extraction from eligible studies including first author, year, study design, number of patients, duration of the study, inclusion criteria, major outcomes, country, average age, gender, ethnicity, results, and therapies. Conflicts among investigators were resolved by discussion with a third author to determine whether there was a conflict among studies.

### Outcome assessment

We assessed the efficacy of associated phenylephrine plus ketorolac injection of 1.0%/0.3% (PE/K) with different control interventions including vehicle, epinephrine, and control in the form of phenylephrine alone. The outcomes of the interest are shown below: pain management as the number of patients on pain medications, opioid medications, and reporting ranges of pain from none to mild. We also evaluated pain and pupil diameter during surgery as aspects of specific ophthalmological outcomes.

### Quality assessment

The Cochrane Handbook (ROB-2) was used for the assessment of RCTs [14]. The quality evaluation encompasses methods such as randomization, allocation concealment, blinding, result data integrity, results of selective reporting, and other potential sources of bias. We used the National Institutes of Health (NIH) tool to assess the quality of case series and cohort studies.

### Statistical analysis

Our statistical analyses in this analysis were performed using the Review Manager (RevMan) software 5.1.4, provided by Cochrane Collaboration. Weighted mean differences with 95% CIs were calculated for continuous data, while pooled Risk ratios with 95% CIs were calculated for dichotomous data. Heterogeneity among the studies was assessed using the I^2^ test. A fixed-effects model was applied if the heterogeneity was low (I^2^ < 50%), whereas a random-effects model was used in cases of moderate to high heterogeneity (I^2^ ≥ 50%). Subgrouping analyses were used to explore heterogeneity among studies and resolve it.

In addition, sensitivity analyses were also conducted to evaluate the heterogeneity of the results by excluding studies with a high risk of bias or varying sample sizes.

## Results

### Literature search

**Fig. 1:**
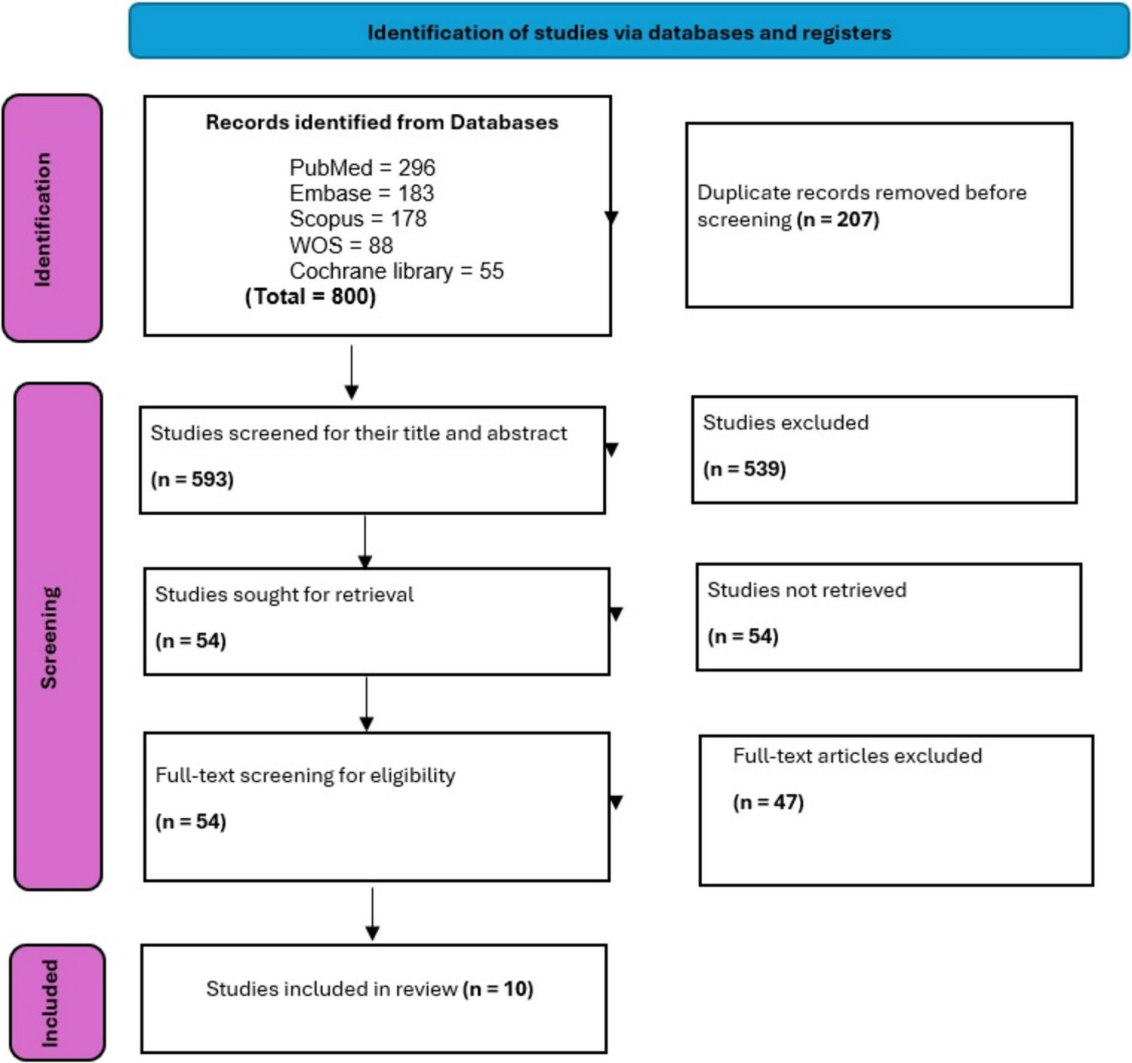
Prisma flow diagram of the included studies

Through the systematic search, we identified 800 articles from several databases, and the duplicated records were removed (*n* = 207). As a result, 593 articles were screened for their title and abstract; 539 studies were not relevant and were excluded. Fifty-four articles went through the full-text screening. After applying the pre-defined criteria, 45 studies were excluded. Ten studies were included in the review. The literature search and screening process is illustrated in the PRISMA flow chart (Fig. 1).

### Characteristics of included studies

Ten studies [2, 5, 10, 15–18] were included in this analysis (8 published and 2 unpublished articles), eight of them were randomized controlled trials [13, 15–18] including the two unpublished articles (NCT02895035 and NCT02132312), one retrospective cohort study (Jackson 2020) [17] and one case series (Silverstein 2018) [2]. All studies were conducted in the United States with a total of 220,061 patients across the ten studies. The study populations had diverse baseline characteristics. Both Wilson et al. and NCT02132312 included pediatric patients with a mean age of 1.2 years, while the remaining studies included adult patients with a mean age of 70.9 years in the remaining eight trials. The majority of the participants were women, comprising 124,957 individuals, which represents 56.78% of the total. The characteristics of the included studies and their results are shown in Supplementary Table 1. As well as baseline characteristics of patients in the included studies are shown in Table 1.

**Table 1:** Baseline characteristics of patients in the included studies

Study ID	Study arms	Age (years), Mean (SD)	Sex (female), N (%)	Race, *N* (%)					
				American Indian or Alaska Native	Native Hawaiian or Other Pacific Islander	Asian	White	Black or African American	Others
Donnenfeld 2017	Vehicle	68.5 (9.6)	33 (57.9%)	0	1 (1.8%)	6 (10.5%)	46 (80.7%)	4 (7.0%)	0
	Phenylephrine	66.8 (8.6)	33 (60.0%)	0	0	7 (12.7%)	46 (83.6%)	1 (1.8%)	1 (1.8%)
	PE/K	66.4 (11.2)	37 (66.1%)	0	0	5 (8.9%)	45 (80.4%)	6 (10.7%)	0
Donnenfeld 2022	Epinephrine	70.1 (7.09)	33(58.9%)	NA	NA	NA	NA	NA	NA
	PE/K	70.1 (7.09)	33(58.9%)	NA	NA	NA	NA	NA	NA
Hovanesian 2015	Placebo	68 (10.2)	237 (58.5%)	1 (0.2%)	0	37 (9.1%)	313 (77.3%)	54 (13.3%)	0
	PE/K	69 (9.4)	236 (58.6%)	2(0.5%)	1 (0.2%)	28 (6.9%)	330 (81.9%)	40 (9.9%)	2(0.5%)
Wilson 2020	Phenylephrine	1.4 (1.2)	19 (49%)	NA	NA	0	30 (77%)	3 (8%)	0
	PE/K	1.0 (1.1)	16 (48%)	NA	NA	1 (3%)	26 (79%)	5 (15%)	1 (3%)
Donnenfeld 2019	PE/K	72.1 (8.8)	22 (53.7%)	NA	NA	NA	NA	NA	NA
	epinephrine	73.5 (7.2)	11 (57.9%)	NA	NA	NA	NA	NA	NA
Jackson2020	PE/K	74.3 (6.67)	2,870 (55.8%)	NA	NA	NA	NA	NA	NA
	NO PE/K	74 (7.41)	121,306 (56.8%)	NA	NA	NA	NA	NA	NA
silverstein2018	PE/K	73.04 (1.45)	NA	NA	NA	NA	23 (92.0%)	1 (4.0%)	NA
	Placebo	74.16 (1.42)	NA	NA	NA	NA	21 (84.0%)	2 (8.0%)	NA
NCT02132312	PE/K	1.0 (1.1)	16 (48.5%)	NA	NA	NA	NA	NA	NA
	Phenylephrine HCl	1.4 (1.2)	19 (48.7%)	NA	NA	NA	NA	NA	NA
NCT02895035	-	73.1 (7.1)	36 (85.7%)	NA	NA	NA	NA	NA	NA
				NA	NA	NA	NA	NA	NA

Abbreviation: *NA* not applicable, *PE/K* phenylephrine and ketorolac, *PE* phenylephrine

### Quality assessment of the included studies

The risk of bias assessment revealed that the included studies were all at low risk of bias, except the Donnenfeld 2019 [5] study which showed a high risk of bias. A summary of the risk of bias assessment domains is shown in (Supplementary Figs. 1 and 2). Moreover, Silverstein et al. and Jackson et al. showed good quality using the NIH tool (Supplementary Tables 2 and 3).

### Outcomes

#### Efficacy outcomes

##### Patients taking any pain medications

**Fig. 2:**
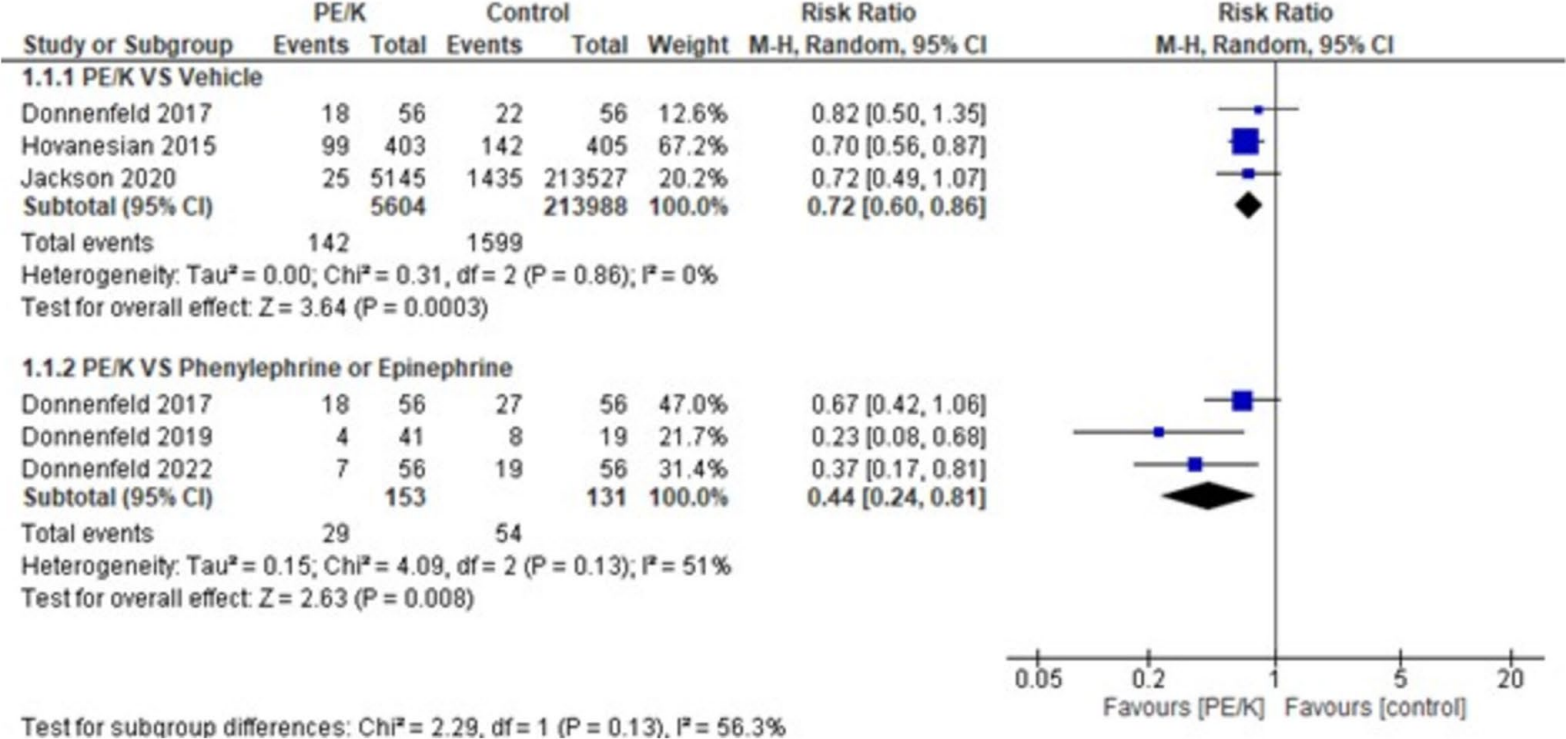
Patients taking any pain medications

When evaluating this outcome in PE/K vs vehicle group & PE/K vs epinephrine and phenylephrine group, the overall effect estimates indicated a statistically significant difference favoring PE/K in both subgroups for PE/K vs vehicle (RR = 0.72, 95% CI: [0.60 to 0.86], *p* < 0.0003). The combined results showed homogeneity (*p* = 0.86, I2 = 0%) and for PE/K and phenylephrine or epinephrine group, the overall effect estimates indicated a statistically significant difference between the two arms favoring PE/K (RR = 0.44, 95% CI: [0.24 to 0.81], *p* < 0.008). The combined results showed heterogeneity (*p* = 0.13, I2 = 51%) with heterogeneity among the subgroups (*p* = 0.13, I2 = 56.3%) (Fig. 2). Furthermore, when evaluating the same outcome with sensitivity analysis results also favored PE/K over vehicle & Epinephrine and Phenylephrine (RR = 0.72, 95% CI: [0.60 to 0.86], *p* < 0.0003), (RR = 0.31, 95% CI: [0.17 to 0.59] respectively, with significant heterogeneity among the subgroups (*p* = 0.01, I2 = 83.3%) after excluding Donnenfeld 2017 [16] (Supplementary Fig. 3).

##### Patients taking any opioid medications

When evaluating taking any opioid medications, the overall effect showed statistically significant results (RR = 0.45, 95% CI: [0.23 to 0.89], *p* < 0.02) and the combined results showed significant heterogeneity (*p* = 0.07, I2 = 63%). The subgroups analyzing PE/K to no PE/K groups showed no significant results (RR = 0.72, 95% CI: [0.49 to 1.07], *p* < 0.11), but when the PE/K compared to the epinephrine group a statistically significant difference was found (RR = 0.31, 95% CI: [0.17 to 0.59], *p* < 0.0003), with significant heterogeneity among the subgroups (*p* = 0.03, I2 = 79.3%). Due to the lack of data from the included studies, it was inapplicable to compare PE/K to the vehicle in this outcome (Supplementary Fig. 4).

##### Patients with no to mild pain

This analysis compares PE/K to the vehicle group and phenylephrine or epinephrine group with an overall RR of 1.37 (95% CI: 1.19, 1.57) and *p* < 0.00001. The combined results showed homogeneity (*p* = 0.66, I2 = 0%), and the test for overall effect is significant. Furthermore, the subgroups analyzing PE/K to the vehicle (RR = 1.51, 95% CI: [1.17 to 1.94], *p* < 0.001) also evaluated the same outcome between PE/K and phenylephrine or epinephrine statistically significant difference between the two arms favoring PE/K (RR = 1.32, 95% CI: [1.11 to 1.55], *p* < 0.001). with significant homogeneity among the subgroups (*p* = 0.37, I2 = 0%) (Supplementary Fig. 5). Moreover, while evaluating this outcome by Sensitivity analysis and excluding Donnenfeld 2022 [18] the overall RR was 1.54 (95% CI: 1.21, 1.95) and *p* < 0.0004. The combined results showed homogeneity (*p* = 0.69, I2 = 0%), the subgroups analyzing PE/K to vehicle (RR = 1.51, 95% CI: [1.17 to 1.94], *p* < 0.001) but for PE/K and phenylephrine or epinephrine (RR = 1.76, 95% CI: [0.88 to 3.52], *p* < 0.11) with significant homogeneity among the subgroups (*p* = 0.69, I2 = 0%) (Supplementary Fig. 6).

##### Patients with severe pain

**Fig. 3:**
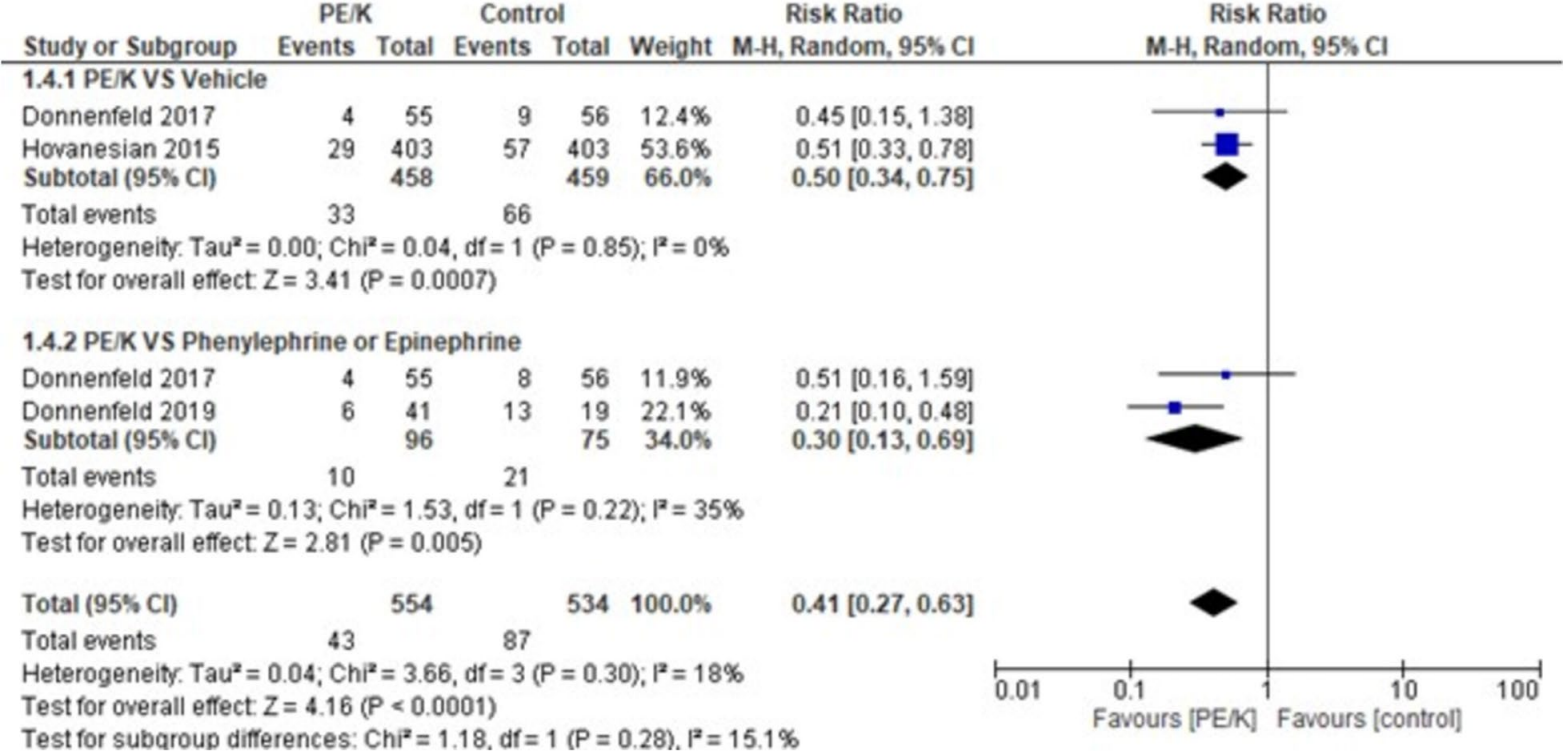
Patients with severe pain

When assessing patients with severe pain, the overall effect estimates showed a statistically significant difference favoring PE/K (RR = 0.41, 95% CI: [0.27 to 0.63], *p* < 0.0001) and the combined results showed minimal heterogeneity (*p* = 0.30, I2 = 18%). Moreover, the subgroups analyzing PE/K vs vehicle and PE/K vs phenylephrine or epinephrine showed significant results favoring PE/K in both subgroups (RR = 0.50, 95% CI: [0.34 to 0.75], *p* < 0.0007) and (RR = 0.30, 95% CI: [0.13 to 0.69], *p* < 0.005) respectively, mild heterogeneity was among the subgroups (*p* = 0.28, I2 = 15.1%) (Fig. 3).

##### Patients with pupil diameter less than 6.0 mm

**Fig. 4:**
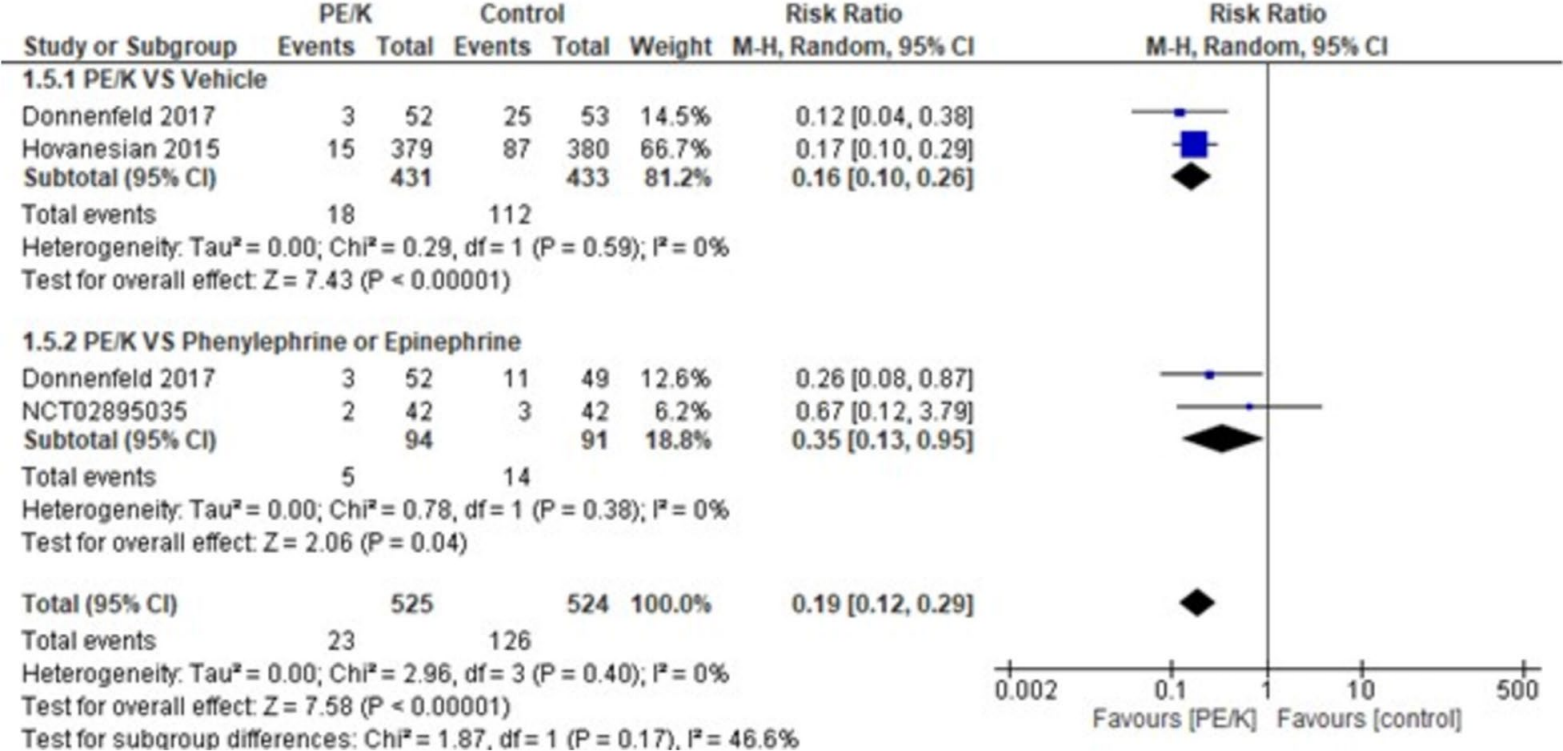
Patients with pupil diameter less than 6.0 mm

When evaluated, PE/K showed statistically significant results (RR = 0.19, 95% CI: [0.12 to 0.29], *p* < 0.00001) and the combined results showed homogeneity (*p* = 0.40, I2 = 0%). the subgroups analyzing PE/K vs vehicle and vs phenylephrine, or epinephrine showed significant results favoring PE/K in both subgroups (RR = 0.16, 95% CI: [0.10 to 0.26], *p* < 0.00001) and (RR = 0.35, 95% CI: [0.13 to 0.95], *p* < 0.03) respectively, moderate heterogeneity was detected among the subgroups (*p* = 0.17, I2 = 46.6%) (Fig. 4).

##### Ocular pain score

**Fig. 5:**
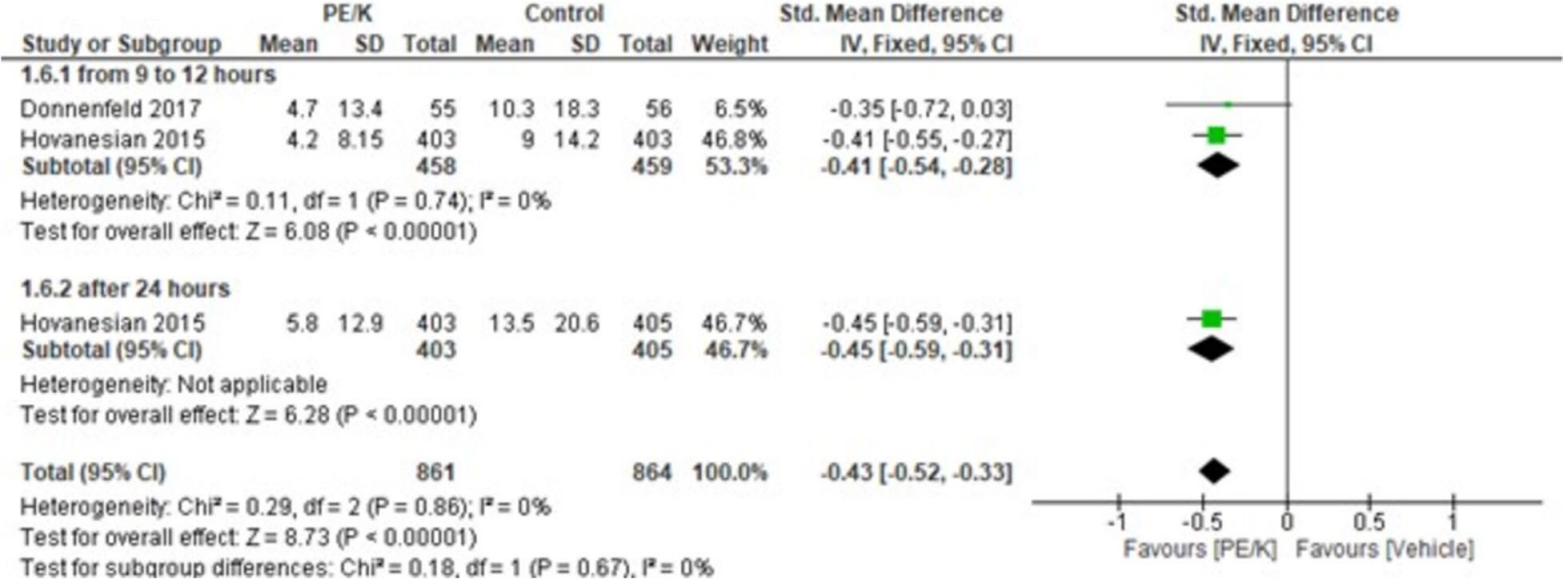
Ocular pain score (PE/K VS Vehicle)

This analysis compares PE/K to the vehicle, epinephrine and phenylephrine, phenylephrine alone and epinephrine alone, as for PE/K to vehicle results showed statistically significant favoring PE/K (MD = −0.43, 95% CI: [−0.52 to −0.33], *p* < 0.00001) and the combined results showed homogeneity (*p* = 0.86, I2 = 0%). Moreover, the subgroups analyzing PE/K after twelve, twenty-four hours showed significant results favoring PE/K over the vehicle in both subgroups (MD = −0.41, 95% CI: [−0.54 to −0.28], *p* < 0.00001), (MD = −0.45, 95% CI: [−0.59 to −0.31], *p* < 0.00001) respectively, homogeneity was detected among the subgroups (*p* < 0.67, I2 = 0%) (Fig. 5). Moreover, PE/K vs epinephrine or phenylephrine showed statistically significant favoring PE/K (MD = −0.81, 95% CI: [−1.08 to −0.53], *p* < 0.00001) and the combined results showed heterogenicity (*p* = 0.17, I2 = 40%), the subgroups analyzing PE/K intraoperatively, 10 min postoperative after twenty-four hours showed significant results favoring PE/K over epinephrine and phenylephrine in subgroups (MD = −0.72, 95% CI: [−1.10 to −0.33], *p* < 0.0002), (MD = −1.05, 95% CI: [−1.52 to −0.57], *p* < 0.0001), (MD = −0.56, 95% CI: [−0.94 to −0.18], *p* < 0.004) respectively, heterogenicity was detected among the subgroups (*p* < 0.29, I2 = 20.2%) (Supplementary Fig. 7).

Furthermore, when assessing PE/K vs phenylephrine the overall effect showed a statistically significant difference favoring PE/K (MD = −0.44, 95% CI: [−0.68 to −0.21], *p* < 0.0002) and the combined results showed homogeneity (*p* = 0.59, I2 = 0%), the subgroups analyzing PE/K after 12 h revealed no significant difference (MD = −0.46, 95% CI: [−0.84 to −0.08], *p* < 0.02), while significant difference was observed after 24 h (MD = −0.43, 95% CI: [−0.74 to −0.13], *p* = 0.005, no heterogenicity was detected among the subgroups (*p* < 0.91, I2 = 0%) (Supplementary Fig. 8). Finally, when PE/K was compared to epinephrine the overall effect showed a statistically significant difference favoring PE/K (MD = −0.81, 95% CI: [−1.08 to −0.53], *p* < 0.00001) and the combined results showed heterogenicity (*p* = 0.17, I2 = 40%), the subgroups analyzing PE/K intraoperatively, 10 min postoperative, after twenty-four hours showed significant results favoring PE/K over Epinephrine in subgroups (MD = −0.72, 95% CI: [−1.10 to −0.33], *p* < 0.0002), (MD = −1.05, 95% CI: [−1.52 to −0.57], *p* < 0.0001), (MD = −0.56, 95% CI: [−0.94 to −0.18], *p* < 0.004) respectively, heterogenicity was detected among the subgroups (*p* < 0.29, I2 = 20.2%) (Supplementary Fig. 9).

##### Change in pupil diameter from baseline till the end of surgery

When evaluating the change in pupil diameter from baseline till the end of the surgery, the overall effect estimates indicated a statistically significant difference favoring PE/K (MD = 0.54, 95% CI: [0.32 to 0.75], *p* < 0.00001) and the combined results showed heterogeneity (*p* = 0.0001, I2 = 71%). When analyzing PE/K vs vehicle it showed a statistically significant difference favoring PE/K (MD = 0.60, 95% CI: [0.52 to 0.68], *p* < 0.00001), on the contrary, PE/K failed to demonstrate a significant difference when compared with epinephrine or phenylephrine (MD = 0.41, 95% CI: [−0.03 to 0.86], *p* = 0.07) (Supplementary Fig. 10). The detected heterogeneity was resolved by applying a leave-one-out sensitivity analysis and excluding NCT02132312 (Supplementary Fig. 11).

##### Best corrected visual acuity (BCVA) log score on day 1

When assessing BCVA log score on day 1 the overall effect estimates indicated insignificant difference between PE/K vs vehicle nor PE/K VS Phenylephrine (MD = 0.04, 95% CI: [−0.05 to 0.14], *p* < 0.38), (MD = 0.00, 95% CI: [−0.10 to 0.10], *p* < 1) respectively, homogeneity was detected among the subgroups (*p* = 0.53, I2 = 0%) (Supplementary Fig. 12).

##### Postoperative ocular inflammation (mean summed ocular inflammation score (SOIS)) on day 1

While assessing this outcome, the overall effect estimates showed a significant difference favoring PE/K over the vehicle and phenylephrine (MD = −0.18, 95% CI: [−0.33 to 0.03], *p* < 0.02) and the combined results showed homogeneity (*p* = 0.36, I2 = 2%). the subgroups analyzing PE/K vs vehicle indicated statistically significant difference favoring PE/K (MD = −0.19, 95% CI: [−0.35 to −0.03], *p* < 0.02), but PE/K vs phenylephrine showed no difference (MD = −0.10, 95% CI: [−0.55 to 0.35], *p* < 0.66, homogeneity was detected among the subgroups (*p* = 0.71, I2 = 0%) (Supplementary Fig. 13).

##### NRS photophobia (severe)

When evaluating this outcome at 6 h and day one results showed no significant difference between PE/K vs vehicle nor phenylephrine, analyzing subgroups at 6 h PE/K vs vehicle (RR = 1.00, 95% CI: [0.32 to 3.15], *p* < 1), and for PE/K vs phenylephrine (RR = 2.04, 95% CI: [0.19 to 21.81], *p* < 0.56), homogeneity was detected among subgroups (*p* = 0.60, I2 = 0%) (Supplementary Fig. 14). Moreover as regarding the results at 1 day, PE/K vs vehicle showed (RR = 0.60, 95% CI: [0.30 to 1.17], *p* < 0.13) and for PE/K vs phenylephrine, it showed (RR = 0.34, 95% CI: [0.07 to 1.61], *p* < 0.17) homogeneity was detected between subgroups (*p* = 0.51, I2 = 0%) (Supplementary Fig. 15).

#### Safety outcomes

For safety outcomes, this study analyzed six different adverse events (elevated intraocular pressure, eye pain, eye inflammation, headache, ocular discomfort, and conjunctival hyperemia):

##### Elevated intraocular pressure

The overall effect estimates indicated no significant difference between PE/K and the other arms (RR = 0.80, 95% CI: [0.33, 1.97]). PE/K vs vehicle (RR = 1.34, 95% CI: [0.70, 2.29], *p* = 0.38), while PE/K vs epinephrine or phenylephrine (RR = 0.80, 95% CI: [0.33, 1.97], *p* = 0.51) (Supplementary Fig. 16).

##### Eye pain

In four studies the overall effect estimates indicated no significant difference between PE/K and the other arms. Analyzing subgroups in PE/K vs vehicle (RR = 0.81, 95% CI: [0.61, 1.08], *p* = 0.15) with mild heterogenicity (*p* = 0.25, I2 = 25%). While for PE/K vs phenylephrine group (RR = 0.78, 95% CI: [0.46, 1.31], *p* = 0.35) with no heterogeneity between subgroups (*p* = 0.70, I2 = 0%) (Supplementary Fig. 17).

##### Eye inflammation

For this outcome, four trials were analyzed and the overall effect estimates indicated no significant difference between PE/K and the other arms, analyzing subgroups in PE/K vs vehicle (RR = 1.31, 95% CI: [0.64, 2.67], *p* = 0.46) with moderate heterogeneity (*p* = 0.14, I2 = 54%). While for PE/K vs phenylephrine group (RR = 1.26, 95% CI: [0.58, 2.74], *p* = 0.56) with no heterogeneity (*p* = 0.49, I2 = 0%) (Supplementary Fig. 18).

##### Headache

The overall effect estimates indicated no significant difference between PE/K and other arms. When analyzing subgroups in PE/K vs vehicle (RR = 0.70, 95% CI: [0.45, 1.10], *p* = 0.12) with homogeneity (*p* = 0.81, I2 = 0%). While for PE/K vs phenylephrine group (RR = 1.93, 95% CI: [0.37, 10.10], *p* = 0.44) (Supplementary Fig. 19).

##### Ocular discomfort

It was clear that there is no significant difference in overall effect estimates between PE/K and other arms, analyzing subgroups in PE/K vs vehicle (RR = 0.52, 95% CI: [0.27, 1.00], *p* = 0.05). Also, for PE/K vs phenylephrine group (RR = 0.96, 95% CI: [0.06, 15.03], *p* = 0.98) (Supplementary Fig. 20).

##### Conjunctival hyperemia

The overall effect estimates indicated no significant difference between PE/K and other arms. When analyzing subgroups in PE/K vs vehicle (RR = 0.66, 95% CI: [0.11, 3.84], *p* = 0.64). While for PE/K vs phenylephrine group (RR = 0.87, 95% CI: [0.17, 4.52] (Supplementary Fig. 21).

## Discussion

The primary objective of this systematic review and meta-analysis was to evaluate the efficacy and safety profile of phenylephrine/ketorolac compared to placebo/vehicle, epinephrine and/or phenylephrine in cataract surgeries. Our findings suggest that the combination of phenylephrine and ketorolac provides significant benefits in terms of maintaining intraoperative mydriasis and reducing postoperative pain without additional risk of adverse events.

One of the key findings was that PE/K was associated with significantly lower postoperative pain and inflammation compared to placebo, epinephrine, and norepinephrine, which aligns with the known anti-inflammatory properties of ketorolac [19]. Additionally, we found that the use of PE/K correlated with less use of pain medications and opioids during the surgery. Previous research has established ketorolac as an effective agent in managing postoperative inflammation and pain in various surgical settings, including ophthalmic surgeries [20, 21]. Advances in surgical techniques and equipment have raised the standard for cataract surgery, including precise outcomes and minimal complications, making effective pain management crucial for patient cooperation and optimal results. Our study supports the use of combination PE/K in cataract surgery to minimize patient discomfort and ensure cooperation to achieve optimum refractive outcomes.

Trauma to tissue during cataract surgery results in the release of prostaglandins (PGs) that mediate the inflammation and pain during and after the surgery. Thus, the observed superiority of PE/K in reducing pain and inflammation postoperatively can be explained by ketorolac’s ability to decrease the levels of PGs by inhibiting both cyclooxygenase-1 (COX-1) and cyclooxygenase-2 (COX-2) [22–24].

Furthermore, our analysis revealed the superior ability of PE/K to maintain intraoperative mydriasis, as well as in preventing intraoperative miosis compared to vehicle, epinephrine, or phenylephrine. Adequate mydriasis is particularly critical for facilitating surgical visualization of the lens and reducing the risk of complications during cataract surgery. Studies have demonstrated that inadequate mydriasis is associated with an increased risk of complications, including posterior capsule rupture and prolonged surgery time [25, 26]. Our results complement these findings, showing that PE/K significantly reduces the risk of these complications by maintaining optimal pupil size throughout the procedure.

The safety profile of PE/K was compared to other alternative regimens in our study by examining six adverse events related to cataract surgery: elevated IOP, eye pain, eye inflammation, headache, ocular discomfort, and conjunctival hyperemia. Overall, the study found no significant differences between PE/K and other treatment arms across all analyzed outcomes, indicating that PE/K has a comparable safety profile to these alternatives.

No significant difference between PE/K and other alternatives in terms of IOP elevation. This finding aligns with previous studies indicating that phenylephrine, often used during cataract surgeries, does not significantly impact IOP in the postoperative period [27, 28]. Similarly, ketorolac’s has not been associated with IOP changes [29, 30].

The findings from three studies involving 1,031 patients showed no significant differences in the incidence of headaches between the PE/K and other arms. Headaches are known to be associated with cataract surgeries, particularly in the immediate postoperative period, and our results suggest that PE/K does not increase this risk compared to other commonly used agents.

Ocular discomfort is a common side effect following cataract surgery, and previous research has highlighted the benefit of using NSAIDs like ketorolac in reducing this discomfort, especially when combined with mydriatic agents like phenylephrine. However, the results of this meta-analysis suggest that PE/K’s benefits in reducing ocular discomfort may be similar to those of alternative treatments.

Lastly, no significant difference was found in conjunctival hyperemia between PE/K and other arms. While ketorolac has anti-inflammatory properties that might reduce hyperemia, the results suggest that PE/K’s effects on this outcome are comparable to those of vehicle and epinephrine. This finding is supported by existing studies that show mixed results regarding the efficacy of NSAIDs in preventing hyperemia postoperatively [31].

Despite the aforementioned promising findings, our study has several limitations. The heterogeneity among the included studies, particularly regarding surgical techniques, dosing regimens, and patient demographics, may have introduced variability into our results. Although subgroup analyses were conducted to mitigate these differences, the potential for residual confounding remains. Additionally, the limited number of trials and small sample sizes for certain adverse events may have affected the power to detect significant differences. Moreover, the number of studies directly comparing phenylephrine/ketorolac with epinephrine and/or norepinephrine was limited, which may affect the strength of our conclusions in this regard.

The observed heterogeneity in the multiple outcomes was best solved by applying sensitivity analysis and subgrouping the included studies based on the time of evaluation, yet it poses significant limitation in generalizing our results to the whole population. This heterogeneity could be attributed to the difference in age, as Wilson et al. 2020 included mainly pediatric patients (mean; 1, SD; 1.1), and there are significant differences in pain perception between this group and adults undergoing cataract surgery. It could also be attributed to a variety of other factors (e.g. surgical techniques, patient demographics, or comorbidities), for instance in both Donnenfeld et al. 2019 and 2022 [5, 18], patients received bromfenac preoperatively, which may have influenced pain responses and introduced some heterogeneity. Additionally, in Donnenfeld et al. (2019), patients underwent femtosecond laser-assisted cataract surgery (FLACS), which could have affected their perception of pain, further contributing to the observed heterogeneity.

The possibility of publication bias should also be considered, as studies with negative or neutral results may not have been published, potentially skewing our analysis, but due to the limited number of the included studies in each outcome (less than 10), publication bias was not applicable as the results would’ve not been accurate and potentially misleading as suggested by the Cochrane Handbook [32].

Future research should address these limitations by conducting large-scale, multicenter randomized controlled trials that directly compare phenylephrine/ketorolac with epinephrine and norepinephrine across diverse patient populations. Such studies should also investigate long-term outcomes, including chronic postoperative inflammation and patient-reported quality-of-life measures. Furthermore, assessing the cost-effectiveness of phenylephrine/ketorolac relative to traditional agents will be crucial for informing clinical practice and healthcare policy, especially in settings with limited resources.

## Conclusion

In conclusion, our systematic review and meta-analysis provide robust evidence supporting the use of phenylephrine/ketorolac in cataract surgeries. The combination offers superior efficacy in maintaining intraoperative mydriasis, reducing postoperative pain, and minimizing inflammation, making it a preferable alternative to placebo, epinephrine, and norepinephrine. These findings suggest that phenylephrine/ketorolac may set a new standard in cataract surgery management, offering the potential for enhanced surgical outcomes and improved patient comfort.

## Supplementary Information

Below is the link to the electronic supplementary material.

Supplementary file1 (DOCX 466 KB)

Supplementary file2 (DOCX 572 KB)
