# Supplementary material for: Optimizing pain management and pupil dilation in cataract surgery: a systematic review and meta-analysis of phenylephrine/ketorolac (OMIDRIA®)
Source: Graefes Arch Clin Exp Ophthalmol. 2025 Mar 29;263(8):2265–75. doi: 10.1007/s00417-025-06811-y (PMC12414053; doi:10.1007/s00417-025-06811-y)
Supplement: Supplementary file 1 — Supplementary file1 (DOCX 466 KB) [file 417_2025_6811_MOESM1_ESM.docx]

**Supplementary Table 1.** Characteristics of the included studies.

| **Study ID** | **Study Design** | **Study arms** | | **Country** | **Number of centers** | **Drugs used during surgery** | **Total Participants** | **Inclusion criteria** | **Primary Outcomes** | **Conclusion** |
| --- | --- | --- | --- | --- | --- | --- | --- | --- | --- | --- |
|  |  | **Intervention** | **Control** |  |  |  |  |  |  |  |
| **Donnenfeld 2017** | randomized double-masked vehicle-controlled trial | phenylephrine and ketorolac injection 1.0%/0.3% | a balanced salt solution(vehicle), ketorolac, and phenylephrine | United States. | 23 | NA | 223 | 1)The patient must be at least 18 years of age or older. 2) has a corrected distance visual acuity (CDVA) of 20/400 or better in the non-study eye. 3)the intraocular pressure (IOP) in the study eye must be between 5 and 22 mmHg. 4)the individual is scheduled to undergo unilateral (single-eye) primary cataract surgery, using a coaxial phacoemulsification device and the insertion of an acrylic intraocular lens (IOL), under topical anesthesia. | a change in pupil diameter during surgery and postoperative ocular pain within 12 hours after surgery. | The drug was found to be safe and effective in maintaining pupil dilation (mydriasis) and reducing postoperative eye pain. Both, ketorolac and phenylephrine, contributed to the therapeutic effects observed. The combination of ketorolac and phenylephrine showed superior efficacy compared to either agent used alone in maintaining an intraoperative pupil diameter of 6.0 mm or larger. |
| **donnenfeld 2019** | prospective, single-masked, comparative study | phenylephrine and ketorolac 1.0%/0.3% | epinephrine | United States. | 1 | 1) 0.5 mL of intracameral preservative free lidocaine 1%  2) phenylephrine and ketorolac 1.0%/0.3% in study group 3) epinephrine (1 mg/mL)in control group  4) intravenous (IV) fentanyl | 60 | Patients who scheduled to undergo cataract surgery, either using femtosecond laser-assisted technology (FLACS) or conventional phacoemulsification techniques under topical anesthesia | pain experienced during the surgery, as measured by mean visual analog scale (VAS) pain scores ranging from 0 (no pain) to 10 (extreme pain) assessed 10 mins postoperatively in the recovery room and (2) the use of IV fentanyl during surgery. | The intraoperative administration of a combination of phenylephrine and ketorolac 1.0%/0.3% resulted in a nearly 80% reduction in the need for intraoperative opioid pain medications during cataract surgery. It also decreased the mean Visual Analogue Scale (VAS) pain scores by approximately 50% and significantly reduced the risk of patients experiencing moderate-to-severe pain during the cataract surgery. |
| **donnenfeld 2022** | randomized, double-masked, self-controlled trial | phenylephrine 1.0%-ketorolac 0.3% | epinephrine | United States. | 1 | 1) 1% no preserved lidocaine  2) 2 mg intravenous midazolam 3) Either P/K or epinephrine  4) Fentanyl | 56 | 1)Patients were required to be between 50 and 80 years of age 2) Patients were scheduled to undergo cataract surgery in both eyes. 3) the patients' insurance coverage of P/K | the proportion of procedures requiring opioid medication (i.e., need for fentanyl) to control pain during surgery | The administration of the phenylephrine and ketorolac (P/K) combination significantly reduced pain and the need for the opioid medication fentanyl during cataract surgery. |
| **hovanesian2015** | Prospective randomized clinical trials. | phenylephrine 1.0%–ketorolac 0.3% | placebo | United States. | 20 | 1) 483 mM phenylephrine and 89 mM ketorolac formulated in 20 mM sodium citrate buffer (study drug) 2) 20 mM sodium citrate buffer (placebo)  3) 0.01% [w/v] phenylephrine and 0.003% [w/v] ketorolac | 808 | 1)Patients must be 18 years of age or older. 2) Patients were scheduled to undergo either cataract extraction and lens replacement, or refractive lens exchange. 3) Patients had a baseline intraocular pressure (IOP) between 5 and 22 mmHg. | (1) intraoperative pupil diameter during surgery (2) ocular pain during the early postoperative period following surgery. | phenylephrine 1.0% and ketorolac 0.3% administration intracamerally (into the anterior chamber of the eye) through the irrigation solution during cataract surgery was safe for the patients and also effective in maintaining adequate pupil dilation (mydriasis) throughout the surgical procedure .This same combination therapy was also effective in reducing the level of postoperative ocular (eye) pain experienced by the patients. |
| **jackson2020** | retrospective cohort study | phenylephrine and ketorolac 1.0%/0.3% | no intervention | United States. | NA | phenylephrine and ketorolac 1.0%/0.3% in 5145 patients | 218672 | 1)Patients be at least 65 years old at the time of their first cataract-related surgical procedure. 2)Patients must have had continuous enrollment in the MarketScan database, which includes both medical and pharmacy benefits, for at least 6 months prior to and 7 days following the index date (the date of the first cataract-related surgery). | the number of patients who filled a prescription for oral opioids on the day of surgery or within the first 2 days post-surgery along with the number of days of dosing supplied and the pill count for the first qualifying prescription | Patients who did not have recent opioid use and received the phenylephrine-ketorolac (P/K) combination during their cataract surgery had a greater incidence of preoperative comorbidities & higher risk for surgical complexity Despite these higher-risk factors, the patients who received the P/K combination were prescribed fewer opioid pills following their cataract surgery, compared to patients who did not receive the P/K treatment. |
| **silverstein2018** | Prospective case series | phenylephrine 1.0%–ketorolac 0.3% injection | basic saline solution | United States. | 1 | 1)topical tropicamide 1.0% 2) phenylephrine hydrochloride 2.5% as well as topical benzocaine  3) Tetracaine was used in select cases | 50 | 1)Men with current or previous use of tamsulosin 2) The patients were scheduled to undergo unilateral (single eye) cataract surgery using the phacoemulsification technique not undergoing any other concurrent ophthalmic (eye) surgical procedure. | pupil diameter at 3 time periods (before cataract extraction, after cataract extraction, after IOL implantation), presence or absence of iris prolapse, and severity of iris billowing (mild, moderate, or severe) | The use of the phenylephrine 1.0% and ketorolac 0.3% combination, when added to the irrigation solution during cataract surgery, led to significant benefits in patients at risk of Intraoperative Floppy Iris Syndrome (IFIS) as Better prevention of pupil constriction (miosis) , reduced pupil billowing ,decreased incidence of iris prolapse .A new grading scale for intraoperative iris abnormalities might be used for future evaluation of this combination therapy. |
| **wilson2020** | Randomized double-masked phase 3 clinical trial. | phenylephrine and ketorolac (PE/K) 1.0%/0.3% | phenylephrine 1.0% | United States. | 17 | 1)4mL of either PE/K 1.0%/0.3% or PE 1.0% was injected into a 500cc bottle of a balanced salt solution | 78 | 1)Patients must be between 0 and 3 years of age at the time of their cataract surgery. 2)Patients must be undergoing cataract extraction, with or without the implantation of an intraocular lens. 3)Informed consent and HIPAA authorization must be provided by a parent or legal guardian, in accordance with local regulations and the requirements of the governing Institutional Review Board (IRB) or Independent Ethics Committee (IEC). 4)The parent or legal guardian must indicate that they understand the study procedures and restrictions, and that they are able, willing, and likely to fully comply with them | the mean change in the pupil diameter over time from surgical baseline (immediately prior to Volume 46 Issue 6 June 2020 surgical incision) to the end of the surgical procedure (wound closure). Intraoperative pupil size was determined by measuring the pupil diameter | The PE/K 1.0%/0.3% combination was a safe treatment option for use in children undergoing cataract surgery. This combination therapy successfully maintained the necessary pupil dilation during the surgical procedure in the pediatric patients. Patients who received the PE/K 1.0%/0.3% combination experienced reduced levels of postoperative pain compared to the control group. |
| **NCT02132312** | A randomized, parallel-group, double-masked, controlled study | phenylephrine and ketorolac 1.0%/0.3% (OMS302) | Phenylephrine HCl | United States. | 11 | NA | 72 | 1. between 0 to 3 years of age at the time of surgery. 2. will have unilateral primary cataract extraction with or without lens replacement. 3. Have informed consent and Health Insurance Portability and Accountability Act (HIPAA) Authorization provided by a parent or legal guardian  4. A parent or legal guardian has indicated that they understand and are able, willing, and likely to fully comply with study procedures and restrictions. | Change From Baseline in Pupil Diameter, Postoperative pain measured using the Alder Hey Triage Pain Score and safety outcomes | NA |
| **NCT02895035** | A randomized, parallel-group, double-masked, controlled study | phenylephrine and ketorolac 1.0%/0.3% | Epinephrine | United States. | NA | NA | 42 | 1. Patients who are older than 18 years of age 2. Patients who are planned to undergo bilateral cataract surgery 3. Patients with baseline IOP of 5 - 22 mm Hg | Change From Baseline in Pupil Diameter, number of Eyes With Pupil Diameter Less Than 6 mm and safety outcomes |  |

**Abbreviation: NA: not applicable - PE/K: phenylephrine and ketorolac – PE: phenylephrine – HIPPA: Health Insurance Portability and Accountability**

**Supplementary Table 2.** NIH for observational cohort.

| Study ID | Case definition Adequate. | Representativeness of the cases | Selection of Controls | Definition of Controls | Comparability based on design or analysis part 1 | Comparability based on design or analysis part 2 | Ascertainment of Exposure | Same method of ascertainment for cases and controls | D9 | Overall |
| --- | --- | --- | --- | --- | --- | --- | --- | --- | --- | --- |
| Jackson 2020 | Low | Low | Low | Low | Low | Low | Some concern | Low | Unclear | Low |

**Supplementary Table 3.** NIH for case series study.

| Criteria | Silverstein 2018 |
| --- | --- |
| 1. Was the study question or objective clearly stated? | Yes |
| 2. Was the study population clearly and fully described, including a case definition? | Yes |
| 3. Were the cases consecutive? | Yes |
| 4. Were the subjects comparable? | Yes |
| 5. Was the intervention clearly described? | Yes |
| 6. Were the outcome measures clearly defined, valid, reliable, and implemented consistently across all study participants? | Yes |
| 7. Was the length of follow-up adequate? | NA |
| 8. Were the statistical methods well-described? | Yes |
| 9. Were the results well-described? | Yes |
| 10. Was there any consideration of confounding factors in the design or analysis? | No |
| 11. Was the funding source or conflict of interest stated? | NA |
