# Supplementary material for: Optimizing pain management and pupil dilation in cataract surgery: a systematic review and meta-analysis of phenylephrine/ketorolac (OMIDRIA®)
Source: Graefes Arch Clin Exp Ophthalmol. 2025 Mar 29;263(8):2265–75. doi: 10.1007/s00417-025-06811-y (PMC12414053; doi:10.1007/s00417-025-06811-y)

**Supplementary Figure 1.** The bias-risk assessment diagram of the included articles**.**
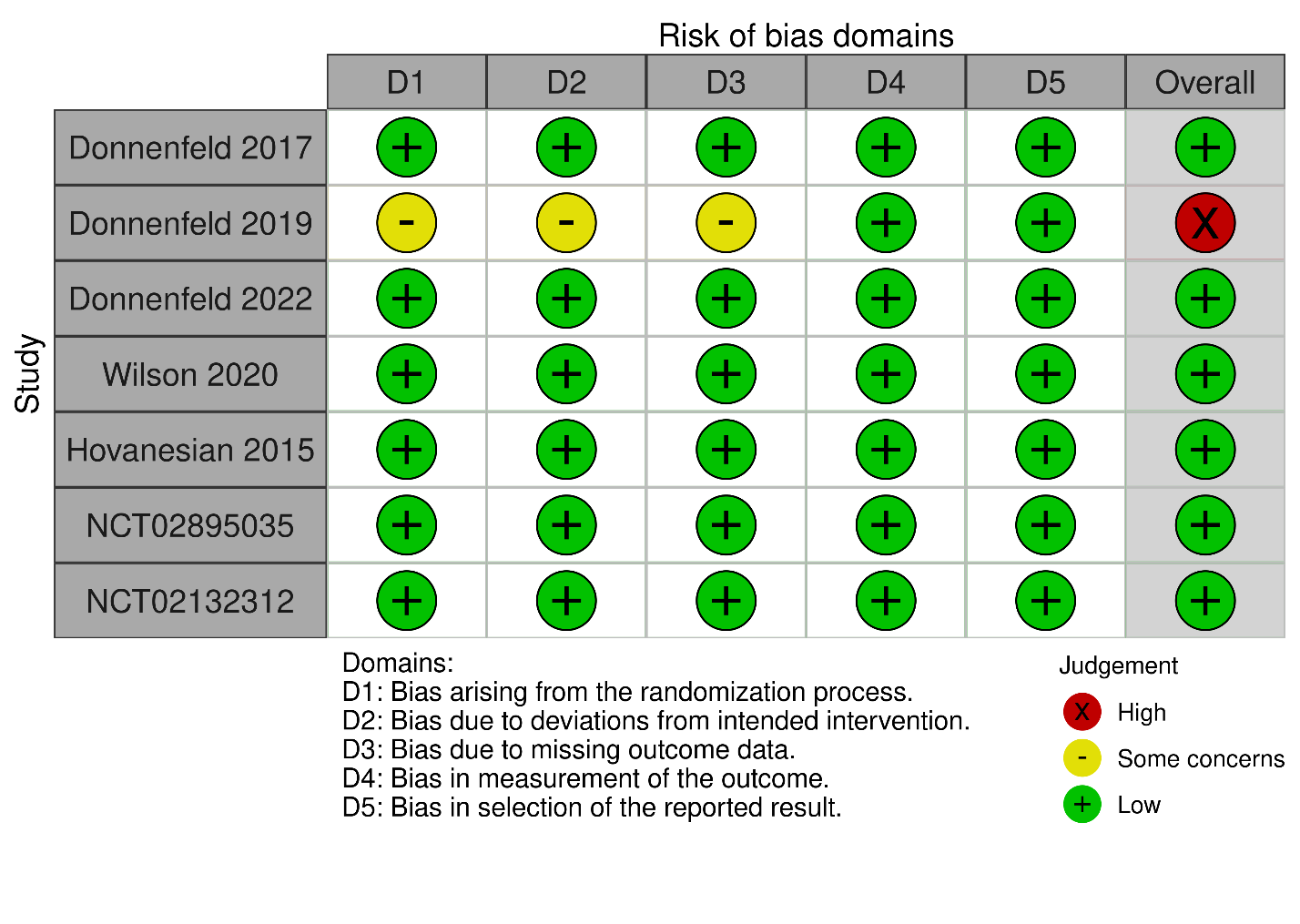


**Supplementary Figure 2.** The bias evaluation bar graph of the included articles.


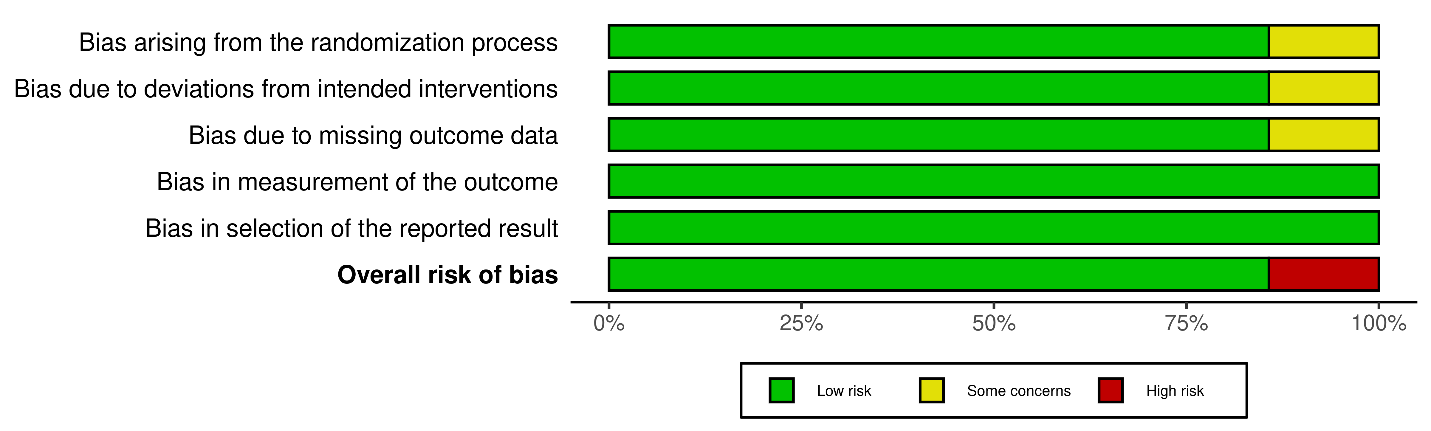


**Supplementary Figure 3.** Patients taking any pain medications (sensitivity analysis).
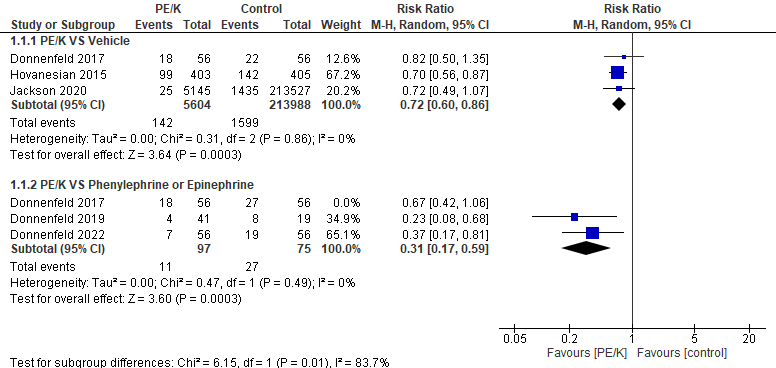


**Supplementary Figure 4.** Patients taking any opioid medications.
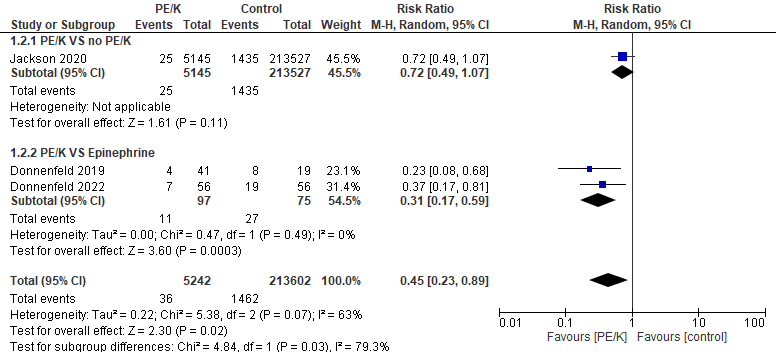


**Supplementary Figure 5.** Patients with no to mild pain.
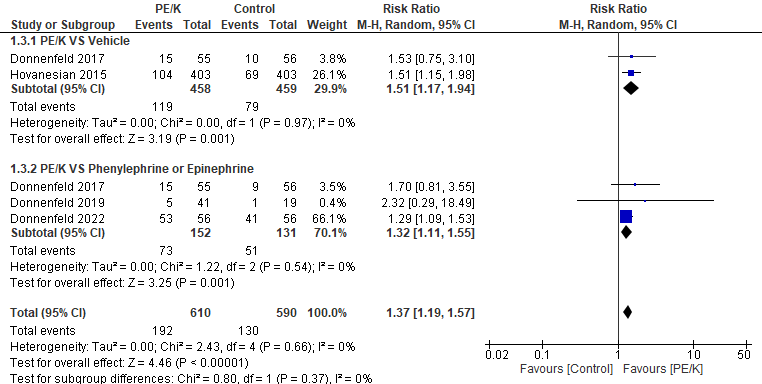


**Supplementary Figure 6.** Patients with no to mild pain (sensitivity analysis)
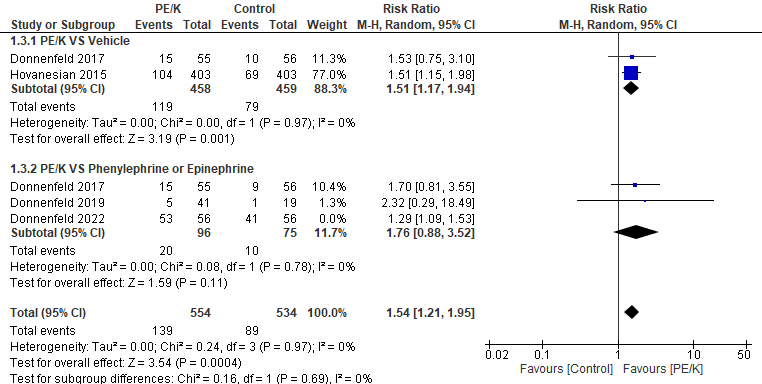


**Supplementary Figure 7.** Ocular pain score (PE/K VS Epinephrine and Phenylephrine).
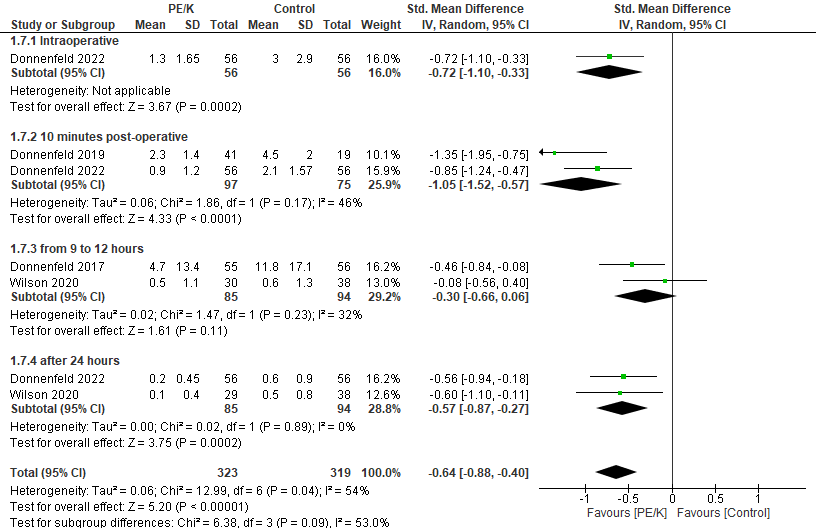


**Supplementary Figure 8.** Ocular pain score (PE/K VS Phenylephrine).
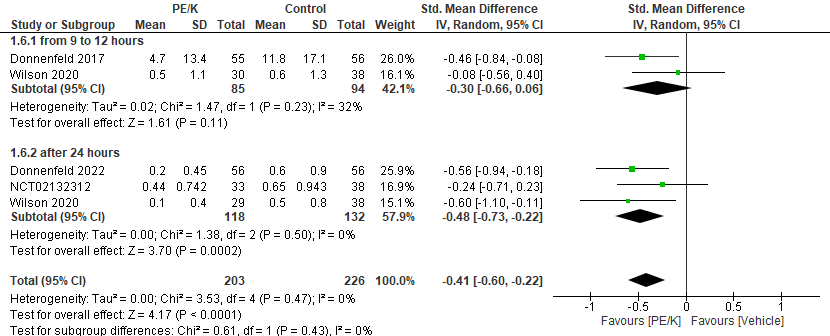


**Supplementary Figure 9.** Ocular pain score (PE/K VS Epinephrine).
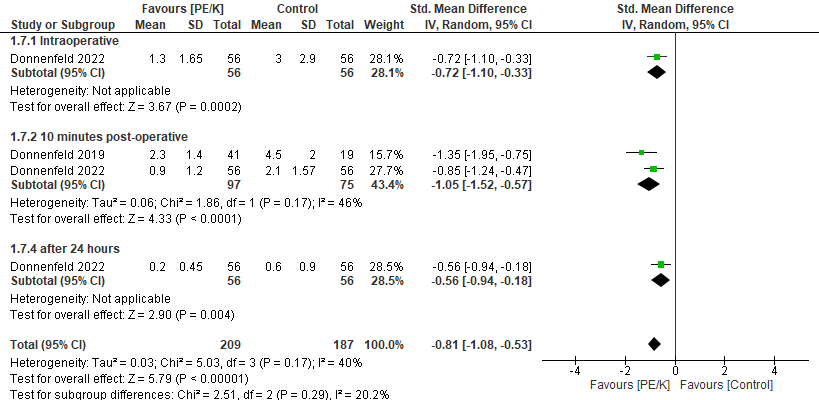


**Supplementary Figure 10.** Change in pupil diameter from baseline till the end of surgery (no sensitivity analysis).
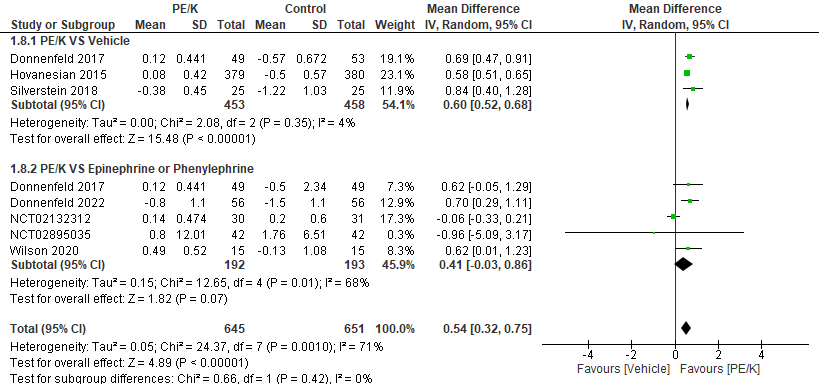


**Supplementary Figure 11.** Change in pupil diameter from baseline till the end of surgery (with sensitivity analysis removing NCT02132312).
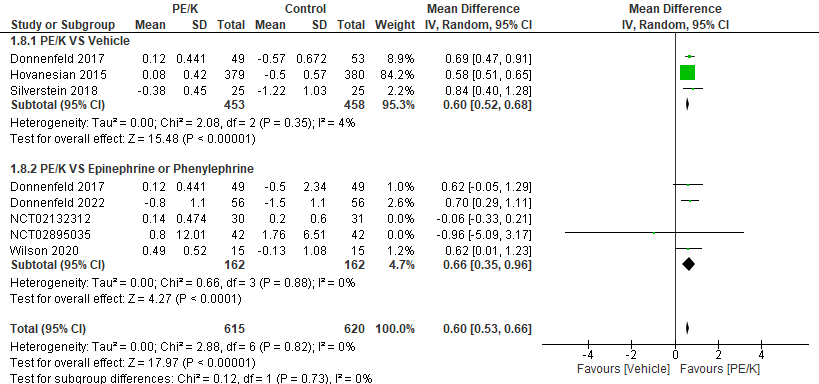


**Supplementary Figure 12.** Best corrected visual acuity (BCVA) log score on day 1.
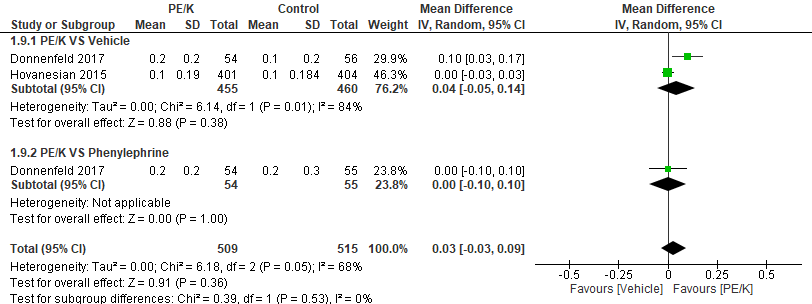


**Supplementary Figure 13.** Postoperative ocular inflammation- Mean Summed Ocular Inflammation Score (SOIS) on day 1.
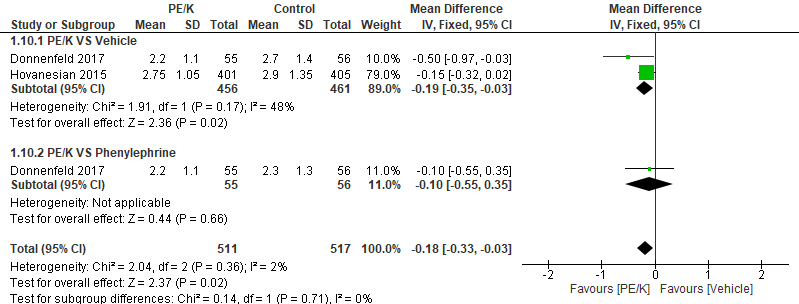


**Supplementary Figure 14.** NRS Photophobia severe at 6 hours.
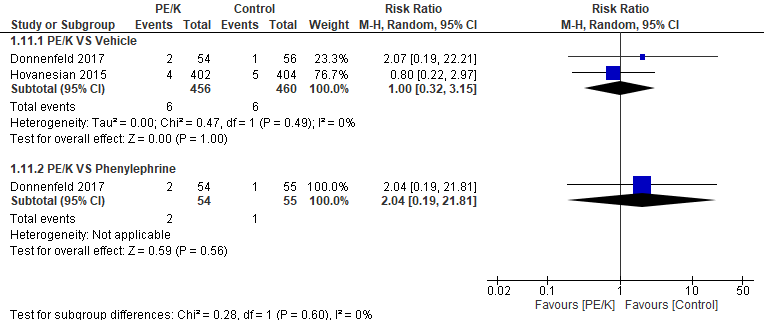


**Supplementary Figure 15.** NRS Photophobia severe at 1 day.
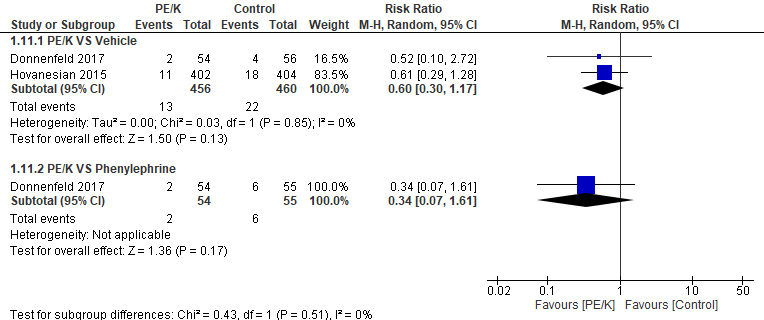


**Supplementary Figure 16.** Elevated intraocular pressure.
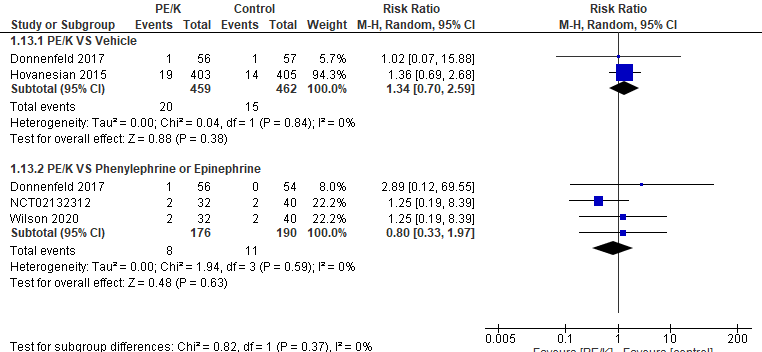


**Supplementary Figure 17.** Eye pain.
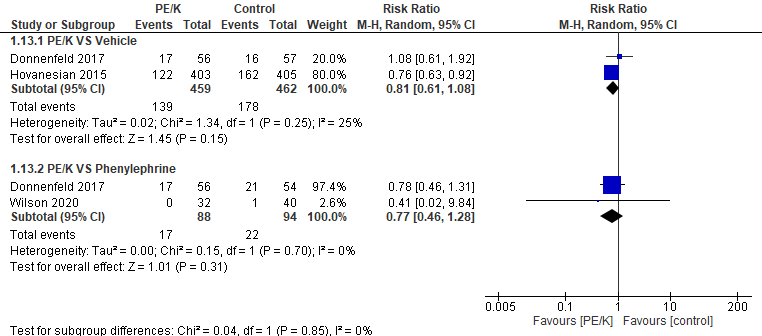


**Supplementary Figure 18.** Eye inflammation.
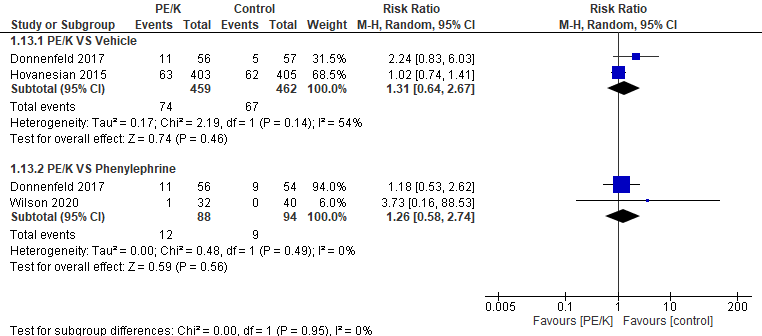


**Supplementary Figure 19.** Headache.
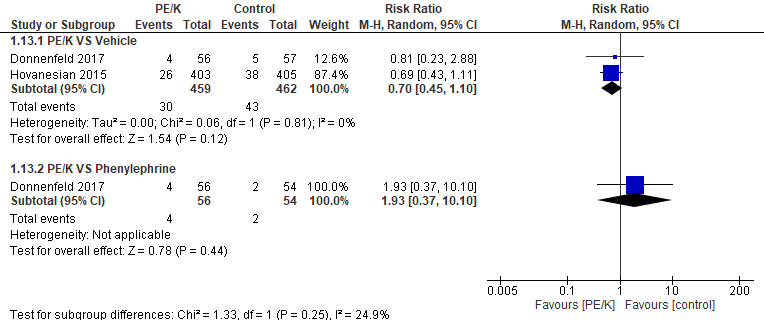


**Supplementary Figure 20.** Ocular discomfort.
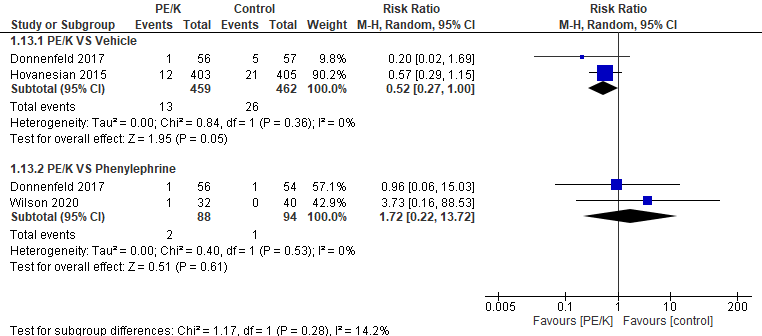


**Supplementary Figure 21.** Conjunctival hyperemia.
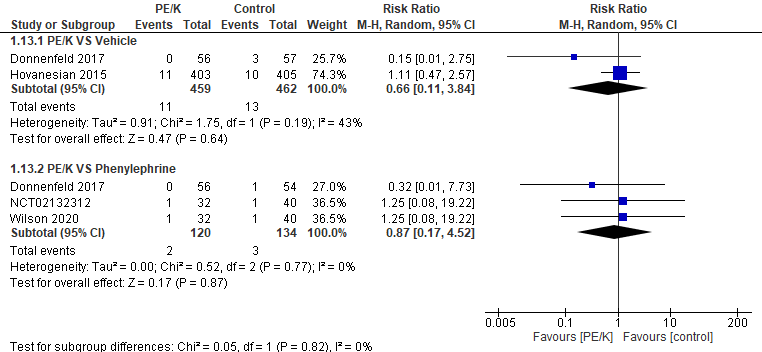

Supplement: Supplementary file 2 — Supplementary file2 (DOCX 572 KB) [file 417_2025_6811_MOESM2_ESM.docx]
